# Gene expression profiling of homologous recombination repair pathway indicates susceptibility for olaparib treatment in malignant pleural mesothelioma in vitro

**DOI:** 10.1186/s12885-019-5314-0

**Published:** 2019-01-30

**Authors:** Sabrina Borchert, Michael Wessolly, Jan Schmeller, Elena Mairinger, Jens Kollmeier, Thomas Hager, Thomas Mairinger, Thomas Herold, Daniel C. Christoph, Robert F. H. Walter, Wilfried E. E. Eberhardt, Till Plönes, Jeremias Wohlschlaeger, Clemens Aigner, Kurt Werner Schmid, Fabian D. Mairinger

**Affiliations:** 1 Institute of Pathology, University Hospital Essen, University of Duisburg-Essen, Essen, Germany; 2 Department of Pneumology, Helios Klinikum Emil von Behring, Berlin, Germany; 3 Department of Pathology, Helios Klinikum Emil von Behring, Berlin, Germany; 4 Department of Medical Oncology, West German Cancer Centre, University Hospital Essen, University of Duisburg-Essen, Essen, Germany; 5 Department of Internistic Oncology, Kliniken Essen Mitte, Essen, Germany; 6 Ruhrlandklinik, West German Lung Centre, University Hospital Essen, University of Duisburg-Essen, Essen, Germany; 7 Department of Thoracic Surgery and Thoracic Endoscopy, Ruhrlandklinik, University Hospital Essen, University of Duisburg-Essen, Essen, Germany; 8 Department of Pathology, Diakonissenkrankenhaus Flensburg, Flensburg, Germany

**Keywords:** Malignant pleural mesothelioma - overall survival, PARP1, BRCAness - BAP1, Olaparib

## Abstract

**Background:**

Malignant pleural mesothelioma (MPM) is a tumour arising from pleural cavities with poor prognosis. Multimodality treatment with pemetrexed combined with cisplatin shows unsatisfying response-rates of 40%. The reasons for the rather poor efficacy of chemotherapeutic treatment are largely unknown. However, it is conceivable that DNA repair mechanisms lead to an impaired therapy response. We hypothesize a major role of homologous recombination (HR) for genome stability and survival of this tumour. Therefore, we analysed genes compiled under the term “BRCAness”. An inhibition of this pathway with olaparib might abrogate this effect and induce apoptosis.

**Methods:**

We investigated the response of three MPM cell lines and lung fibroblasts serving as a control to treatment with pemetrexed, cisplatin and olaparib. Furthermore, we aimed to find possible correlations between response and gene expression patterns associated with BRCAness phenotype. Therefore, 91 clinical MPM samples were digitally screened for gene expression patterns of HR members.

**Results:**

A BRCAness-dependent increase of apoptosis and senescence during olaparib-based treatment of BRCA-associated-protein 1 (*BAP1*)-mutated cell lines was observed. The gene expression pattern identified could be found in approx. 10% of patient samples. Against this background, patients could be grouped according to their defects in the HR system. Gene expression levels of Aurora Kinase A (*AURKA*), *RAD50* as well as DNA damage-binding protein 2 (*DDB2*) could be identified as prognostic markers in MPM.

**Conclusions:**

Defects in HR compiled under the term BRCAness are a common event in MPM. The present data can lead to a better understanding of the underlaying cellular mechanisms and leave the door wide open for new therapeutic approaches for this severe disease with infaust prognosis. Response to *Poly (ADP-ribose)-Polymerase* (*PARP*)-Inhibition could be demonstrated in the *BAP1*-mutated NCI-H2452 cells, especially when combined with cisplatin. Thus, this combination therapy might be effective for up to 2/3 of patients, promising to enhance patients’ clinical management and outcome.

**Electronic supplementary material:**

The online version of this article (10.1186/s12885-019-5314-0) contains supplementary material, which is available to authorized users.

## Background

Malignant pleural mesothelioma (MPM) is a highly aggressive tumour arising from the pleural cavities [1, 2]. Despite treatment, MPM patients have poor prognosis with a median survival of approximately 12 months [3–5]. The state-of-the-art systemic treatment of unresectable and advanced MPM is chemotherapy with a combination of cisplatin and pemetrexed [6, 7]. However, even with aggressive treatment approaches, recurrence or progression occurs in most cases as a result of chemotherapy resistance [6, 8, 9].

The term “BRCAness” is defined as a defect in double-strand break repair (DSBR) of DNA by the homologous recombination repair (HRR) pathway [2, 10].

**Fig. 1:**
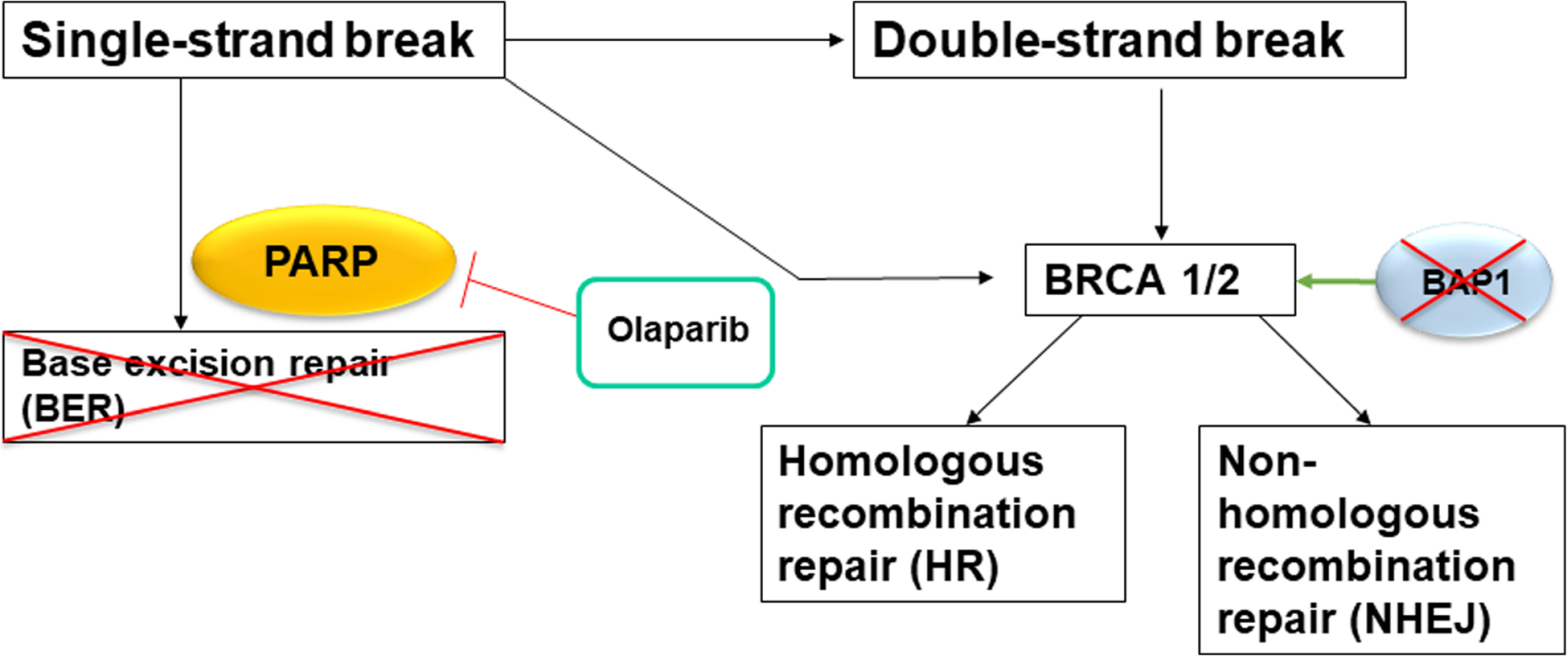
Enhanced base excision repair, due to defective HRR that is caused by BRCAness phenotype, increases the reliance on *PARP1*. It is suggested that loss-of-function mutation of *BAP1* also results in BRCAness phenotype. Inhibition of *PARP1* prevents the alternative repair pathway and thus could lead to apoptosis of the cell

HRR is involved in the repair of DNA lesions blocking the replication fork or inducing double-strand breaks (DSBs) [10]. Alterations in various genes, associated to BRCAness phenotype, were assessed in several tumours. Interestingly, *BAP1* loss-of-function mutation has been found in 26–64% of MPMs [11]. BRCAness leads to genomic instability and therefore could make the tumour more susceptible to different chemotherapeutics targeting these features [12, 13]. Furthermore, alternative repair mechanisms could overcome the lack of HRR and tumour cells could evade apoptosis. *Poly (ADP-ribose)-Polymerase* (*PARP*) is essential for base excision repair (BER) and non-homologous end joining (NHEJ) and may be a target to inhibit alternative repair mechanisms in case of BRCAness [14] (Fig. 1).

*PARP*-inhibitors are already FDA approved drugs for treatment of several cancers with Breast Cancer 1 (*BRCA1*) or *BRCA2* mutations [15]. We hypothesize, that not *BRCA1/2* mutations are exclusively necessary for efficient *PARP*-inhibition therapy. We hypothesize, that *PARP*-inhibitors might also be effective in cancers with alterations in related genes of the whole homologous recombination repair pathway. In addition, a combination of platinum-based chemotherapy with *PARP-*inhibitors is assumed to be more effective than *PARP*-inhibitors alone [12].

The present study was designed to

1. Find predictive gene expression patterns to olaparib treatment, based on HRR key players. Analyse different human MPM cell lines for the presence of defects in HRR pathway. Furthermore, we investigated if MPM cell lines shows sensitivity against *PARP*-inhibition. In this case, we also aimed to find gene expression patterns predictive for treatment with *PARP*-inhibitors.
2. Define patients with altered HRR based on the results of the MPM cell lines analysed in 1) and validate the potential predictive gene expression pattern associated with response to *PARP*-inhibitors in clinical specimens.
3. Investigate associations between patients’ survival prognosis and response to cisplatin, respectively, in association with HRR key players. Based on this study, a preselection of patients with a beneficial response to olaparib treatment might be done.

## Methods

### Study design

#### Finding predictive gene expression patterns, based on HRR key players

We investigated the response of MPM cell lines and lung fibroblasts to treatment with pemetrexed, cisplatin and olaparib. The control lung fibroblast cell line (MRC-5) and MPM cell lines: MSTO-211H, NCI-H2052 and *BAP1*-mutant NCI-H2452 were used for the cell culture experiments. Either single agent olaparib, cisplatin and pemetrexed, or cisplatin in combination with either olaparib or pemetrexed, was added to the cells. Response to treatment was assessed by using three luminescent-based assays detecting apoptosis, necrosis and senescence of cell lines. In addition, we aimed to find possible correlations between response and gene expression patterns associated with BRCAness phenotype.

#### Finding patients with apparent predictive expression patterns

For determination of BRCAness phenotypes, RNA of the cell lines and 90 formalin-fixed, paraffin-embedded (FFPE) patient samples, was isolated and used for digital gene expression analysis of genes listed in Table 1. Correlation tests were used to determine response associated patterns in cell lines. These results were compared with the gene expression pattern of the patient cohort.

**Table 1:** List of genes for gene express ion analysis. The short name and full name of the genes as well as their function are listed [40]

Gene	Official Full Name	Function (40)
*BAP1*	BRCA1 Associated Protein 1	Promotes DSB repair
*BRCA1*	Breast Cancer 1	Cell cycle checkpoints activation, resection of 5′ ends of the DSB, necessary for RAD51 function
*BRCA2*	Breast Cancer 2	Localizes RAD51 to the DSB
*PARP1*	Poly (ADP-Ribose) Polymerase 1	Initiates SSB repair
*RAD51*	RAD51 Recombinase	RAD 51 activity allows DNA to invade homologous double helix serving as template
*RAD50*	RAD50 double strand break repair protein	Forming of the MRN complex
*ATM*	ATM serine/threonine kinase	Detection of DSB
*ATR*	ATR serine/threonine kinase	Detection of DSB
*PALB2*	Partner and localizer of BRCA2	Localizes BRCA2 to the DSB
*BARD1*	BRCA1 associated RING domain 1	Binding partner of BAP1
*EMSY*	BRCA2 interacting transcriptional repressor	Transcription regulator that interacts with BRCA2
*DDB2*	Damage specific DNA binding protein 2	Required for DNA binding in DNA damage repair
*BACH1*	BTB domain and CNC homolog 1	Transcriptional regulator that acts as repressor or activator
*MRE11*	MRE11 homolog, double strand break repair nuclease	Forming of the MRN complex
*NBN*	nibrin	Forming of the MRN complex
*AURKA*	Aurora kinase A	Kinase in cell-cycle, is involved in microtubule formation and stabilization at spindle pole during chromosome segregation
*FANCD2*	Fanconi anaemia complementation group D2	Involved in HRR, is monoubiquinated in response to DNA damage
*BRIP1*	BRCA1 interacting protein C-terminal helicase 1	Involved in HHR by interaction with BRCA1
*CHEK2*	Checkpoint kinase 2	Cell cycle checkpoint regulator and putative tumour suppressor
*RPA1*	Replication protein A1	Activates ATR
*GUSB*	Glucuronidase beta	Normalization for digital expression analysis
*POLR1B*	RNA polymerase I subunit B	Normalization for digital expression analysis
*TUBB*	Tubulin beta class I	Normalization for digital expression analysis
*PGK1*	Phosphoglycerate kinase 1	Normalization for digital expression analysis

#### Investigation of associations between survival prognosis of patients and response to cisplatin

We aimed to find associations between defects in HRR and response to cisplatin using survival prognosis with the help of the results of the digital gene expression analysis (Table 1).

### Patient cohort

#### Specimens

90 FFPE specimens from MPM patients were collected from the Department of Pathology, Helios hospital Emil von Behring (Berlin, Germany) (46 specimens) and of the Institute of Pathology, university hospital Essen (Germany) (44 specimens)

#### Tumour classification

Tumour classification is based on the WHO classification of tumours guidelines (2015) [16]. TNM-staging is based on the Union internationale contre le cancer (UICC) classification of malignant tumours [17]. All samples were confirmed by two experienced pathologists (JW, TM).

#### Eligibility criteria

The study included MPM patients, treated at the West German Cancer Centre or the West German Lung Centre (Essen) between 2006 and 2009 and the Helios Hospital Emil von Behring (Berlin) between 2002 and 2009. Inclusion criteria were the availability of sufficient tumour material and the case to be listed in the clinical registry for tumour response and survival with a complete set of data concerning follow-up and treatment. Each patient underwent first-line chemotherapy regimen consisting of cisplatin and pemetrexed.

The study was conducted retrospectively to identify gene expression-based biomarkers. It was approved by the institutional ethics review board (Ethics Committee of the Medical Faculty of the University Duisburg-Essen, identifier: 14–5775-BO). The investigations conform to the principles of the declaration of Helsinki.

### Clinical and pathological data

Response to chemotherapy was determined radiologically according to modRECIST [18]. Response was classified as complete response (CR), partial response (PR), stable disease (SD) or progressive disease (PD). Remission was classified as CR or PR vs. SD or PD. Progression was classified as CR or PR or SD vs. PD. Progression-free survival was calculated from start of treatment until first radiological progression (modRECIST). Overall survival was determined from initial diagnosis until death or loss of follow up. Collection of all specimens was performed prior to systemic treatment. Surveillance for this study was stopped on August 31, 2014. General patient data are summarized in Table 2.

**Table 2:** Summary of general statistical patient data

Number of Patients	
Histology	
Biphasic	7
Epithelioid	73
Sarcomatoid	6
Unknown	5
Age [Years]	
Minimum	34.57
Median	64.86
Mean	64.26
Maximum	81.76
Unknown	4
Time to death [month]	
Minimum	0.77
Median	17.33
Mean	21.69
Maximum	81.73
Unknown	4
Clinical outcome	
Alive	9
Dead	78
Unknown	4

### Cell culture

MPM cell lines MSTO-211H (biphasic subtype, pemetrexed-sensitive) and NCI-H2052 (epithelioid subtype, cisplatin-sensitive) as well as the cell line NCI-H2452 (*BAP1*-mutant, sarcomatoid subtype) were cultured in Roswell Park Memorial Institute (RPMI) -1640 medium (Thermo Fisher Scientific, Massachusetts), USA. The human lung-fibroblast cell line MRC-5 was used as control cell line. MRC-5 cells were cultured in Minimal Essential medium (Thermo Fisher Scientific). All culture media were supplemented with 10% foetal calf serum and 1% penicillin and streptomycin (Thermo Fisher Scientific).

### Treatment of MPM cell lines with cytostatic agents cisplatin and Pemetrexed or with *PARP*-inhibitor Olaparib

For the treatment of cells, 5000 cells/well were used. The concentrations of the agents were 0.25 μM for pemetrexed (Selleckchem, Houston, USA) and 10 μM for cisplatin (Selleckchem). To evaluate the most efficient concentration for olaparib (Selleckchem), a dilution series comprising 0.1, 0.5, 1, 5, and 10 μM was applied. In addition to single-agent-treatment, cisplatin was combined with pemetrexed as well as 0.5 μM, 1 μM and 5 μM of olaparib to identify synergistic effects.

### Cell state analysis

5000 cells per reaction were applied to detect apoptosis, senescence and necrosis. All reactions were measured using a luminometer (Glo Max Multi + Detection System; Promega).

Senescence was analyzed using the CellTiter-Glo® Luminescent Cell Viability Assay kit (Promega, Wisconsin, USA). 10 μl of Digitonin (30 μg/ml), added to the cells in a separate well 15 min before cell lysis, served as positive control to measure a decrease of cellular viability of 100%.

Necrosis was analysed using the CytoTox-Glo® Assay kit (Promega). Ionomycin (Selleckchem) was used for positive control. Two hours before measurement, 50 μl of Ionomycin (100 μM), was added to the cells in a separate well. After adding 50 μl of the AAF-Glo® reagent to each well, cells were incubated for 15 min at room temperature, protected from light.

The apoptotic potential of the cells was analysed using the Caspase-Glo® 3/7 Assay (G8093, Promega). 100 μl of required cells/well were placed into a white 96-well plate. Staurosporine (10 μM, Selleckchem) served as positive control and was given to the cells in a separate well 4 h before measurement. After adding 100 μl of Caspase-Glo® reagent to each well, cells were incubated for 30 min at room temperature.

Changes in cell state were calculated as percentage of signal gained by the positive control normalized to the baseline (untreated cells).

### RNA isolation from eukaryotic cells and FFPE patient samples using the automated Maxwell system

RNA-isolation of 1 × 10^6^ cells per sample was performed by using the Maxwell purification platform with appertaining reagents (Maxwell RSC simplyRNA Cells Kit, Promega).

RNA-purification of FFPE specimens was performed by using the Maxwell RSC RNA FFPE Kit (Promega).

The concentration of RNA was determined via fluorometric quantification (Qubit, Thermo Scientific) using the RNA Broad range assay kit according to the manufacturer’s instructions. 1 μl of each isolated RNA sample was applied for measurement.

### Gene expression analysis using the NanoString PlexSet assay

The multiplexed digital gene expression assay (PlexSet, NanoString, Seattle, USA) was used for the investigation of BRCAness phenotype gene expression patterns.

PlexSet assay was performed according to the manufacturer’s instructions. 150 ng FFPE derived RNA from patients suffering from MPM and 70 ng of RNA freshly isolated from cell lines MRC-5, MSTO-211H, NCI-H2052 and NCI-H2452 were applied. Hybridization reaction was executed for 18 h. nCounter Prep-Station processing was performed using the high-sensitivity protocol. Cartridges were scanned on the Digital Analyzer (NanoString) with maximal sensitivity (555 fields of view (FOV)). Investigated genes in samples with counts < 100 are considered as not expressed.

### Statistical analysis

For statistical and graphical analyses, the R statistical programming environment (v3.2.3) was used.

Nanostring data processing has been performed as described previously [19, 20]. In detail, Nanostring counts for each gene underwent technical normalization, based on positive controls included in a code set. Subsequently, biological normalization has been performed by calculating a normalization factor for each sample out of the geometric mean of the included mRNA reference genes.

Additionally, all counts with *p* > 0.05 after one-sided t-test versus negative controls plus 2x standard deviations were interpreted as not expressed to overcome basal noise.

Statistical analysis has been performed as described elsewhere [21]. For exploratory data analysis of dichotomous variables either the Wilcoxon Mann-Whitney rank sum test (non-parametric) or two-sided students t-test (parametric) was applied. For ordinal variables with more than two groups, either the Kruskal-Wallis test (non-parametric) or analysis of variance (ANOVA) (parametric) was used to detect group differences.

Double dichotomous contingency tables were analysed using Fisher’s Exact test. For more than two groups, the dependency of ranked parameters was calculated by using the Pearson’s Chi-squared test. Correlations between metric variables were tested by using the Spearman’s rank correlation test as well as the Pearson’s product moment correlation coefficient for linear modelling.

To further specify the different candidate pattern, each unsupervised and supervised clustering to overcome commonalities as well as principal component analysis to overcome differences were performed.

For the assessment of associations between gene expression and progression-free survival (PFS) or overall survival (OS), Kaplan-Meier analysis was performed. Significant differences in PFS or OS between tested groups were determined by using the COXPH-model. Therefore, the Wald-test, likelihood-ratio test and the Score (log rank) test were used.

*P*-values were adjusted by using the false discovery rate (FDR) with a subsequently defined level of statistical significance of *p* ≤ 0.05.

## Results

### Treatment of MPM cell lines with cytostatic agents

The human lung-fibroblast cell line MRC-5, *BAP1*^*wt/wt*^ MPM cell lines MSTO-211H and NCI-H2052, as well as the *BAP1* mutant MPM cell line NCI-H2452 were analysed for apoptosis, senescence and necrosis during treatment with pemetrexed, cisplatin and olaparib.

**Fig. 2:**
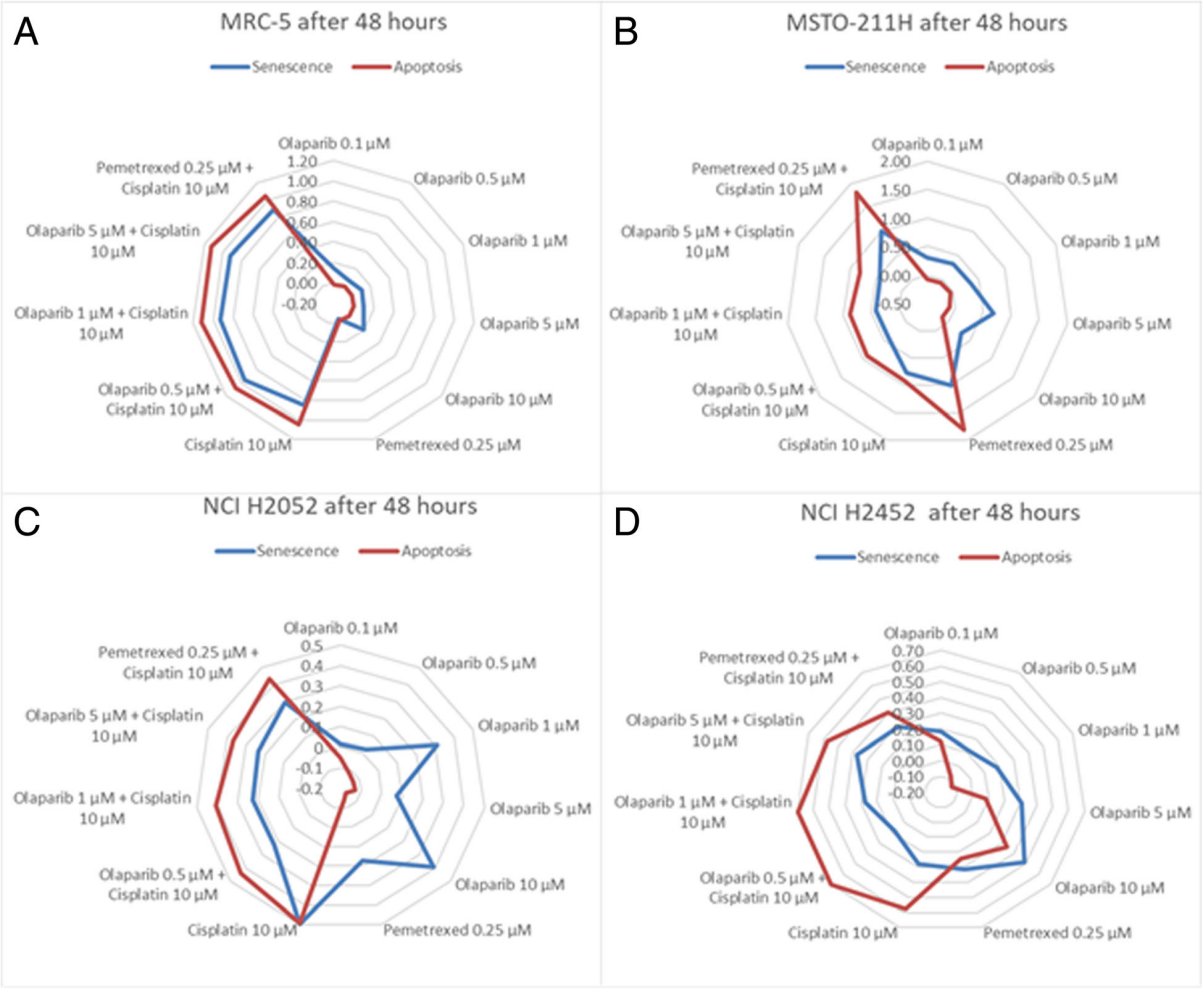
Senescence and apoptosis rate of cell lines after 48 h of incubation. **a:** The effect of both senescence and apoptosis is comparative in MRC-5. **b:** In MSTO-211H, the sharp increase of apoptotic effect of 181% of pemetrexed alone and in combination with cisplatin is illustrated, while senescence showed an effect of 100%. The well treated with 10 μM olaparib showed a senescence effect of 80%, while no apoptotic effect was detected. **c:** NCI H2052 cells showed apoptotic effects only in wells treated with cisplatin or in combination with pemetrexed or olaparib and senescence of 30–50%. Wells treated with 1 μM and 10 μM olaparib showed 40% of senescence, while no apoptotic effect was detected. **d:** NCI H2452 cells showed 70% of apoptosis and only 20% of senescence in wells treated with 0.5 μM, 1 μM or 10 μM olaparib combined with cisplatin. 10 μM Olaparib alone showed 15% higher senescence than apoptosis

While cell lines MRC-5 and MSTO-211H showed a strong induction of apoptosis and senescence by treatment with cisplatin or pemetrexed, olaparib did not have any apoptotic effect on these cells. NCI-H2052 and NCI-H2452 cells showed a notably lower induction of apoptosis than MRC-5 or MSTO-211H cells. The treatment of NCI-H2052 cells with 1 μM and 10 μM single-agent olaparib showed 30–50% induction of senescence. In *BAP1*-mutant NCI-H2452, 0.5 μM and 1 μM olaparib combined with 10 μM cisplatin induced the highest apoptotic effect with low induction of senescence (Fig. 2).

No induction of necrosis could be observed with neither of the used agents, except for the MSTO-211H cells (data not shown). Especially, wells treated with pemetrexed as single agent or in combination showed a necrotic effect.

### BRCAness mRNA marker profiling

#### Differences in gene expression patterns

**Fig. 3:**
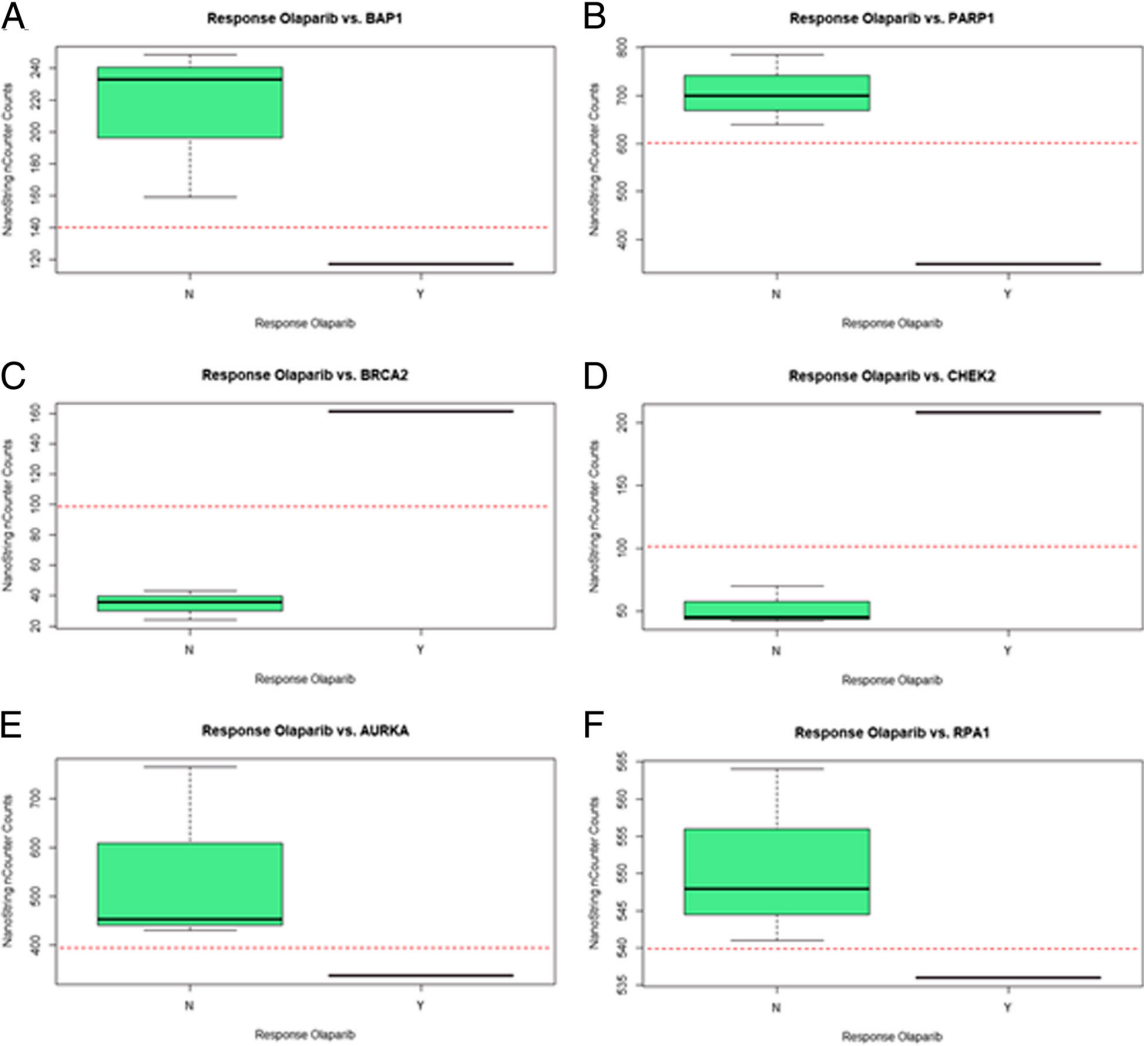
Comparison of significant differences in gene expression level between cell lines with respect to response to treatment with olaparib. Red dotted lines were placed and may represent thresholds between gene expression patterns leading to response to olaparib treatment or not. **a:** *BAP1* was rarely expressed in the cell line that showed response to olaparib treatment (NCI H2452), while it was expressed in other cell lines that showed no response (160 to 250 counts). Threshold was set at 140 counts. **b:** *PARP1* was expressed in NCI H2452 with 350 counts, but other cell lines showed significantly higher expression patterns with 640–785 counts. Threshold was set at 600 counts. **c/d:** *BRCA2* and *CHEK2* are expressed in the cell line that showed response to olaparib (NCI H2452), while no expression was detected in cell lines that showed no response. Thresholds were set at 100 counts for *BRCA2* and *CHEK2*. **e/f:** *AURKA* and *RPA1* are more expressed in cell lines that showed no response, than in NCI H2452. Thresholds were set at 400 counts for *AURKA* and 540 counts for *RPA1*

Differences in gene expression patterns of each cell line with respect to response during olaparib-treatment were observed. The only cell line showing response to olaparib was the *BAP1*-mutant NCI-H2452. *AURKA*, *replication protein A1* (*RPA1*), *BAP1* and *PARP1* were significantly lower or not expressed in NCI-H2452, compared to other cell lines (Fig. 3 a, b, e and f). *BRCA2* and *checkpoint kinase2* (*CHEK2*) were expressed in NCI-H2452, while other cell lines showed no expression with counts < 100 (Fig. 3 c and d).

The expression pattern of these genes in *BAP1*-mutant NCI-H2452 cells could be found in approximately 10% of patient samples (Additional file 1: Table S1).

#### Correlation between samples

**Fig. 4:**
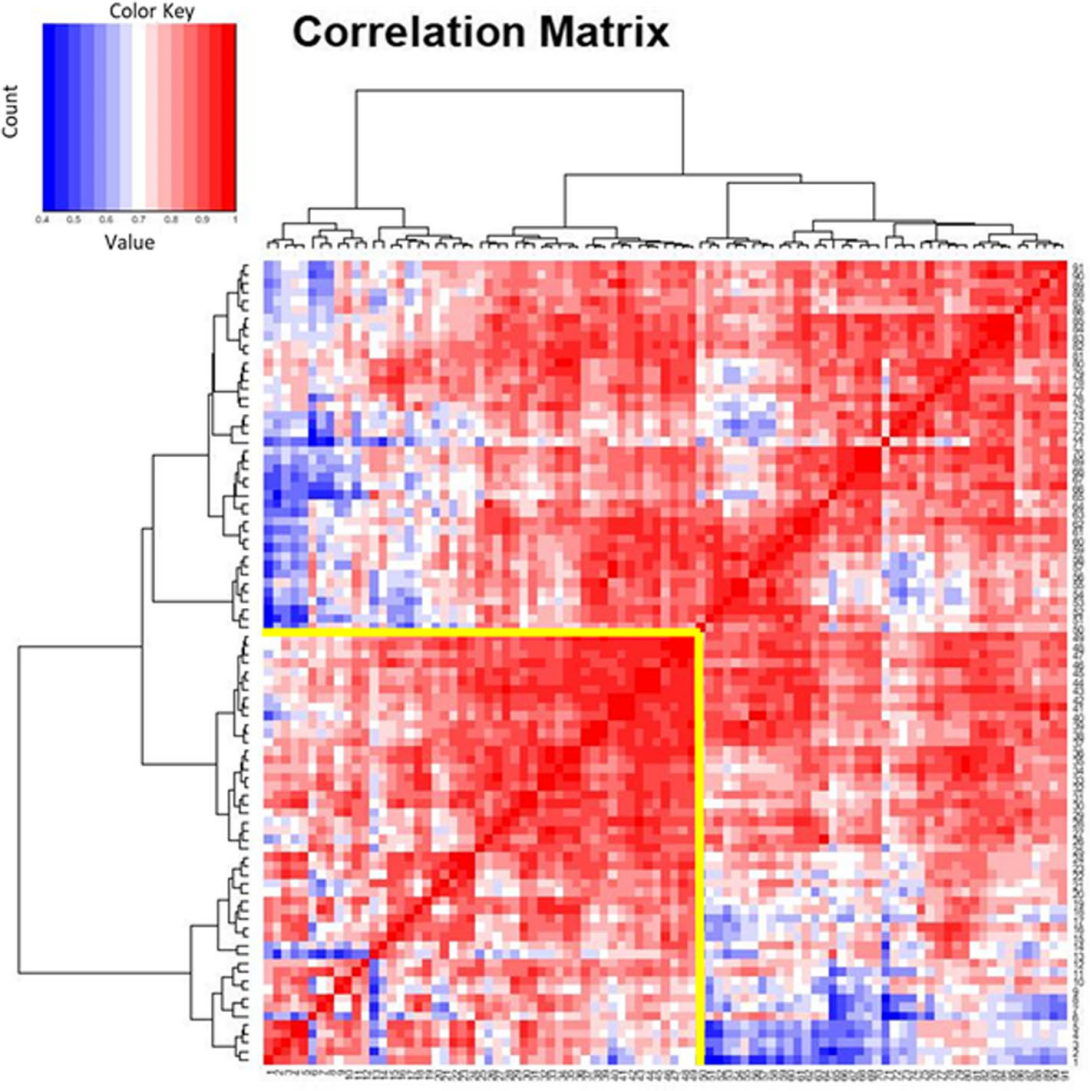
Overall correlation pattern between samples. Unsupervised clustering revealed two distinct groups. The group in the yellow box includes all samples with altered HRR. This box also includes expression patterns of olaparib-responsive NCI-H2052 as well as *BAP1*-mutated NCI-H2452 cells

Overall correlation pattern between different MPM samples is based on Pearson product moment correlation. Unsupervised clustering revealed two distinct groups, each comprising about half of all patients (Fig. 4). One group includes all samples with altered HRR (yellow box), including olaparib-responsive NCI-H2052 as well as BAP1-mutated NCI-H2452 cells. Of note, these 49 samples showed similar gene expression pattern of key enzymes involved in formation of BRCAness-like cell state, overall indicating two different phenotypes of MPM with respect to HRR based repair.

The histomorphology of patient tumours revealed no significant differences in gene expression patterns (Additional file 2: Figure S1).

#### Association with therapy response and survival

**Fig. 5:**
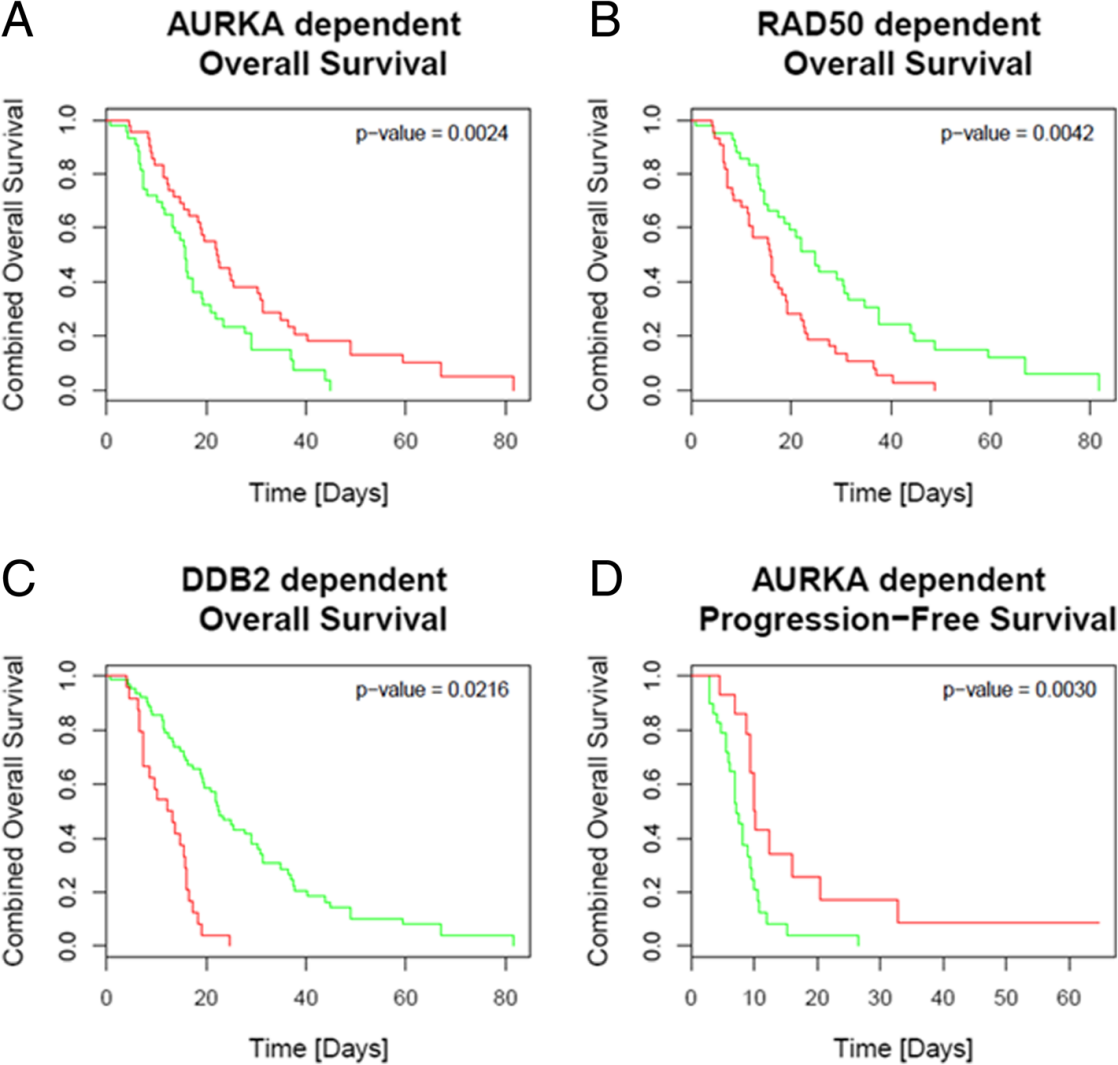
Overall and progression-free survival dependent on gene expression of *AURKA*, *RAD50* and *DDB2*. Low expression of *AURKA* and high expression of *RAD50* and *DDB2* resulted in prolonged overall survival (A-C). Low expression of *AURKA* (D) also resulted in prolonged progression-free survival with *p* < 0.0030. Low expression is highlighted in red, high expression is highlighted in green. The median was used to set a cut-off between high and low gene expression

By determining associations between gene expression and therapy response due to survival, *AURKA*, *RAD50* and damage specific *DNA-damage-binding-protein-2* (*DDB2*) were significantly associated to overall survival with FDR-adjusted *p*-values ≤0.02 (Additional file 3: Table S2). Patients with low expression of *AURKA* have a 2.4-fold higher chance of prolonged overall survival, while high expression of *RAD50* and resulted in 2.3-fold higher risk of dying from their disease. High expression of *DDB2* in patients showed a 4.373-fold chance of prolonged survival. In addition, low expression of *AURKA* showed also a 2.3-fold higher chance of prolonged progression-free survival (Fig. 5).

## Discussion

This study was designed to evaluate gene expression of BRCAness related genes in MPM and their impact on susceptibility to olaparib.

### BRCAness is a common event in MPM

Defects in individual genes that modulate HRR, compiled under the term “BRCAness”, has been found in various tumours [10]. Studies revealed, that impaired HRR results in enhanced use of NHEJ for DSB repair [10, 22–24]. NHEJ repair is more error-prone compared to HRR, often leading to DNA mutations, especially by deletions [10, 25]. This genomic instability predisposes cancer susceptibility caused by BRCAness [26].

In the MPM patients investigated, ten of the 24 tested genes necessary for intact HRR were not significantly expressed (Table 3). Additionally, to this lack of expression, a loss-of-function of three of those genes could already be determined by Betti et al., who also found deleterious mutations in *BRCA1*, *BRCA2* and *PALB2* gene loci [27]. The authors described *BRCA1* and *PALB2* exhibiting a deletion, leading to a nonsense mutational effect, while *BRCA2* showed a frameshift due to deletion [27].

**Table 3:** Tested genes that showed no or very low expression (counts < 100) in MPM patients. FANCD2 showed partially basal expression with a maximum of 214 counts

*AURKA*	*BRCA1*	*CHEK2*	*BRIP1*	*FANCD2*
*BARD1*	*BRCA2*	*EMSY*	*PALB2*	*RAD51*

The loss-of-expression of *RAD51* is not surprising, as it is commonly deleted in malignant mesothelioma due to frequent losses from 15q11.1–22 [28]. Nevertheless, as central element of HR by mediating the invasion into the homologous double helix of the sister chromatid and thereby directly regulated binding partner of *BRCA2*, *RAD51* plays an important role for the shaping of BRCAness.

Beside these main key-players of BRCAness phenotype development, genes of the Fanconi Anaemia (FA) pathway play an important role within this context of DSB repair. Of note, Fanconi anaemia complementation group D2 (FANCD2) as a key member within the FA, shows only basal gene expression levels in all analysed patient samples, indicating an impaired function of the FA pathway. It directly interacts with BRCA1 and BRCA2 but also ataxia telangiectasia mutated (ATM), thereby promoting DNA repair by HR [26, 29, 30]. In contrast, Røe et al. found an overexpression of FANCD2 in microarray data of malignant pleural mesothelioma patients [31], but the analysed sample size of five mesothelioma tissues is quite small. Therefore, these contradictory findings should be examined more detailed.

The correlation matrix of gene expression patterns indicates a cluster of patient samples showing features similar to the olaparib-sensitive NCI-H2452 as well as NCI-H2052 cell lines. It could be assumed, that these patients could benefit from treatment with *PARP*-inhibitors. Assuming a favourable chance of response.

### BRCAness is a strong prognostic factor in MPM

In association with clinicopathological data, high expression of *AURKA* was associated with shortened overall survival and poor prognosis. The high *AURKA* expression pattern possibly explains the high mitotic and proliferative activity, rather found in more aggressive tumours with higher risk of metastatic spread.

In contrast, high expression of *DDB2* and *RAD50* is significantly associated with prolonged survival. *DDB2* is involved in DNA damage repair and is modulated by *BRCA1* and/or p53 [32]. It might be suggested, that *DDB2* together with CHEK1, activates *p53* and thereby triggers *TP53*-induced apoptosis and senescence in response to DNA damage [20]. Barakat et al. showed, that overexpression of *DDB2* enhances cisplatin-sensitivity in ovarian cancer cells [33]. The present data indicate that these observations in ovarian cancer cells could also enhance cisplatin-sensitivity in MPM, resulting in 4.373-fold higher chance of prolonged overall survival.

Zhang et al. demonstrated likewise, that loss of *RAD50* is a key marker of BRCAness in ovarian cancers (OvCa) [34]. Cell culture analysis demonstrated, that loss of *RAD50* augmented OvCa cell’s response to cisplatin and *PARP*-inhibitors [34].

Olaparib induces superior apoptotic response in *BAP1*-mutant tumours in vitro*.* It is shown that *BAP1* is commonly mutated in MPM and thereby might cause defects in HRR as well as a BRCAness phenotype of affected cells [1, 35–37]. We hypothesized, that the *PARP*-inhibitor olaparib could have a therapeutic effect on *BAP1*-mutant MPM and, furthermore, that a combination with cisplatin enhances the inhibitory effect of the former one.

A distinct expression pattern of investigated genes could be found in NCI-H2452 cells. In this cell line, induction of apoptosis could be proven during olaparib-based treatment. Furthermore, we could confirm enhanced therapeutic effects using olaparib in combination with cisplatin. Response of treated NCI-H2452 cells to olaparib has also been shown by Srinivasan et al. [11].

Necrosis was not detected in treated cells, which is preferable, because necrosis leads, compared to apoptosis, to adverse complications in therapy.

The benign human fibroblast cell line MRC-5 was used as benign control. They derive from the same cotyledon (mesoderm), like malignant pleural mesothelioma cells. Therefore, it was preferred, as the e.g. control cell line MET-5a was SV40-immortalized and therefore shows altered culture performance [38]. The TERT1-immortalized cell line LP-9 would be another option for using as control cell line [39]. However, the use of this cell line as control has to be investigated and validated.

### Response of Olaparib on MPM patients might be predicted by similar expression patterns compared to responsive NCI-H2452 cells

Gene expression pattern of NCI-H2452 showed weak correlations to other cell lines, suggesting that gene expression pattern of *BAP1* is associated with enhanced response to *PARP*-inhibitor olaparib. Furthermore, *AURKA*, *RPA1*, *PARP1*, *BRCA2* and *CHEK2* showed significantly different gene expression pattern compared to other cell lines. Transferred to patient samples, approximately 10% of patients have the same gene expression pattern as NCI-H2452 cells, making them suspicious for susceptibility to olaparib based treatment approaches. The *BAP1* mutational status in combination with certain gene expression patterns seem to contribute significantly to cellular response to olaparib. Therefore, this may be a promising therapeutic approach for a substantial portion of MPM patients, especially when combined with platin-based agents.

## Conclusions

In conclusion, response could be demonstrated during treatment of *BAP1*-mutant NCI-H2452 cells with olaparib in combination with cisplatin. Thus, this combined therapy might be effective for up to 2/3 of patients suffering from MPM.

Investigation of BRCAness related genes in MPM patients showed similarities in gene expression patterns compared to MPM cell lines (*BACH1*, *FANCD2* and *RAD51)*, particularly *BAP1*-mutant NCI-H2452 (*BAP1*, *PARP1*, *BRCA2*, *CHEK2*, *AURKA* and *RPA1*). These gene expression patterns represent a novel and promising tool for the prediction of response to the *PARP*-inhibitor olaparib.

## Additional files

Additional file 1: **Table S1.** Normalized counts of genes measured by the Digital Analyzer. (PDF 343 kb)

Additional file 2: **Figure S1.** Expression of tested genes is independent of histomorphology of MPM. The boxplot shows no significant differences between gene expression patterns due to biphasic (B), epithelioid (E), or sarcomatoid (S) MPM. (PDF 59 kb)

Additional file 3: **Table S2.** AURKA, RAD50, and DDB2 showed statistically significant dependencies on overall and progression-free survival. (PDF 33 kb)

## Abbreviations

°C: Degree Celsius
AAF: Alanin-Alanin-Phenylalanin-Aminoluciferin
ADP: Adenosine diphosphate
Anova: Analysis of variance
ATM: Ataxia telangiectasia mutated
ATR: ATM and RAD3-related
BAP1: BRCA1-associated protein 1
BARD1: BRCA1 associated RING domain 1
BER: Base excision repair
BRCA1/2: Breast Cancer 1/2
BRIP1: BRCA1 interacting protein C-terminal helicase 1
CART: Classification and regression tree algorithm
CHEK2: Checkpoint kinase 2
CR: Complete response
CTree: Conditional interference tree
DMSO: Dimethyl sulfoxide
DNA: Deoxyribonucleic acid
DPBS: Dulbecco’s Phosphate Buffered Saline
DSB: Double-strand break
DSBR: Double-strand break repair
FANCD2: Fanconi anaemia complementation group D2
FDA: Food and Drug Administration
FDR: False discovery rate
FFPE: Formalin-fixed paraffin-embedded
FOV: Fields of view
h: Hours
HCF-1: Host cell factor-1
HRR: Homologous recombination repair
min: minutes
MPM: Malignant pleural mesothelioma
MRE11A: Meiotic recombination 11 homologue A
NBS1: Nijmegen breakage syndrome 1
NER: Nucleotide excision repair
Ng: Nanogram
NHEJ: Non-homologous end joining
OS: Overall survival
PALB2: Partner localizer of BRCA2
PARP1: Poly (ADP-ribose) polymerase 1
PD: Progressive disease
PFS: Progression-free survival
PR: Partial response
RLU: Relative light units
RNA: Ribonucleic acid
ROC: Receiver operating characteristic
RPMI: Roswell park memorial institute
SD: Stable disease
SV40: Simian virus 40
UICC: Union international contre le cancer
μl: Microliter
μM: Micro molar
