# Supplementary material for: Gene expression profiling of homologous recombination repair pathway indicates susceptibility for olaparib treatment in malignant pleural mesothelioma in vitro
Source: BMC Cancer. 2019 Jan 30;19:108. doi: 10.1186/s12885-019-5314-0 (PMC6354412; doi:10.1186/s12885-019-5314-0)
Supplement: Supplementary file 1 — Table S1. Normalized counts of genes measured by the Digital Analyzer. (PDF 343 kb) [file 12885_2019_5314_MOESM1_ESM.pdf]

Suppl. Table 1: Normalized counts of genes measured by the Digital Analyzer.

Sample	*ATM*	*ATR*	*AURKA*	*BACH1*	*BAP1*	*BARD1*	*BRCA1*	*BRCA2*	*BRIP1*	*CHEK2*	*DDB2*	*EMSY*	*FANCD2*	*MRE11*	*NBN*	*PALB2*	*PARP1*	*RAD50*	*RAD51*	*RPA1*
**1**	385	289	24	522	536	67	38	2	18	30	305	141	49	96	266	69	549	534	6	448
**2**	1438	631	17	572	428	92	31	19	59	64	230	210	242	166	358	82	1303	591	6	395
**3**	372	337	32	615	523	85	23	5	50	32	346	79	50	70	284	55	834	461	11	257
**4**	238	166	29	486	234	43	20	4	34	35	127	93	55	74	271	42	611	393	10	237
**5**	196	139	31	327	195	64	14	2	37	5	242	52	47	73	242	44	702	548	16	352
**6**	385	325	50	363	227	80	41	15	69	40	221	92	92	117	279	45	700	569	5	203
**7**	533	330	73	443	373	56	51	15	111	72	473	137	128	103	268	177	726	496	29	321
**8**	379	286	42	431	296	34	28	4	61	64	154	85	84	91	226	33	569	655	13	242
**9**	360	187	45	460	383	89	23	14	52	48	364	124	70	94	286	37	510	403	28	386
**10**	192	127	60	555	283	57	25	7	86	60	345	99	34	46	288	61	437	378	33	280
**11**	135	172	74	335	100	80	38	1	120	59	145	69	84	28	252	55	391	250	38	238
**12**	804	299	67	778	331	97	47	29	61	83	160	150	85	123	308	50	1216	426	23	336
**13**	169	202	79	325	148	90	43	7	111	58	173	81	72	65	255	82	315	227	53	316
**14**	415	231	73	434	546	72	39	16	59	48	335	131	71	198	305	45	531	371	36	372
**15**	365	247	83	594	276	93	59	3	115	62	343	158	148	179	477	76	546	363	45	417
**16**	342	237	106	429	312	131	61	8	72	48	393	124	93	181	271	57	549	324	79	472
**17**	462	336	130	394	229	133	88	10	164	85	191	86	147	135	305	63	673	555	36	413
**18**	416	360	195	568	542	108	50	21	105	43	329	87	79	184	332	56	560	307	53	279
**19**	312	341	28	790	366	77	33	3	47	40	286	153	78	173	524	34	766	531	22	311
**20**	587	238	28	372	134	72	20	5	46	46	354	131	63	212	206	43	638	663	21	427
**21**	389	325	47	581	888	75	31	2	42	52	529	133	53	267	373	70	563	513	23	488
**22**	415	255	10	366	70	26	4	7	22	99	562	83	58	302	274	64	398	817	8	346
**23**	654	389	22	628	906	79	16	3	13	36	296	183	19	253	394	53	614	487	3	487
**24**	418	343	42	543	551	66	26	15	101	67	237	139	69	135	240	40	349	374	32	214
**25**	495	345	33	437	400	79	35	14	83	58	259	132	105	171	283	54	459	446	26	335
**26**	296	241	28	298	322	51	18	7	46	49	127	76	65	111	191	40	584	855	15	223
**27**	144	375	7	228	516	3	2	2	112	18	188	93	130	99	226	35	491	1060	5	377
**28**	338	617	45	264	562	34	22	1	47	79	292	93	69	163	348	72	387	986	4	296

Sample	*ATM*	*ATR*	*AURKA*	*BACH1*	*BAP1*	*BARD1*	*BRCA1*	*BRCA2*	*BRIP1*	*CHEK2*	*DDB2*	*EMSY*	*FANCD2*	*MRE11*	*NBN*	*PALB2*	*PARP1*	*RAD50*	*RAD51*	*RPA1*
**29**	402	402	15	654	483	66	11	1	29	85	349	229	74	197	371	30	425	656	1	463
**30**	367	290	53	308	287	82	27	1	161	68	234	115	90	149	354	53	355	634	34	410
**31**	507	467	56	472	369	91	30	22	119	42	352	162	135	166	359	75	598	550	19	420
**32**	362	469	15	403	573	50	3	3	91	15	414	105	39	150	268	64	303	830	3	351
**33**	357	711	4	386	1199	66	4	4	49	49	199	153	49	132	411	36	520	891	4	378
**34**	328	279	57	271	70	87	30	9	259	39	218	81	141	125	200	56	353	766	26	154
**35**	296	173	98	438	280	95	41	9	139	67	151	96	88	88	327	51	444	485	50	283
**36**	718	428	37	333	231	72	36	3	210	87	406	116	120	152	306	47	604	683	22	342
**37**	134	391	109	335	421	59	42	9	45	48	254	56	93	81	304	31	382	243	3	193
**38**	278	310	66	439	358	50	49	18	91	50	213	52	151	147	272	45	547	693	22	392
**39**	287	350	43	367	503	75	38	2	75	88	121	71	90	138	374	32	581	475	17	434
**40**	208	234	122	369	435	64	31	12	125	42	290	123	54	112	225	50	484	441	16	248
**41**	216	360	75	488	123	139	73	2	225	28	320	91	164	136	388	46	827	447	30	462
**43**	238	268	49	363	275	81	34	12	95	45	210	101	121	140	324	42	628	568	7	232
**44**	178	272	59	419	131	47	25	7	93	74	433	66	163	91	288	42	317	491	3	231
**45**	960	953	56	461	716	110	37	14	121	163	361	236	266	258	419	68	964	636	10	297
**46**	265	309	41	295	357	40	33	5	56	37	169	80	90	144	296	48	472	603	11	419
**47**	123	258	38	368	217	47	14	1	48	36	303	32	66	62	193	23	371	371	10	178
**48**	122	247	41	412	346	47	21	12	66	36	125	97	43	147	211	73	528	553	14	293
**49**	266	583	18	471	156	54	4	6	61	56	418	104	73	216	311	51	290	964	2	175
**50**	519	491	16	429	126	46	18	7	33	73	312	89	111	280	360	71	312	888	1	341
**51**	266	235	122	484	415	93	70	34	62	67	92	80	114	155	222	38	384	238	64	285
**52**	555	384	49	521	293	78	27	1	61	78	336	102	119	277	250	55	432	768	9	265
**53**	405	287	60	225	203	83	48	8	36	42	206	92	79	154	218	47	523	372	19	276
**54**	386	395	51	475	408	121	34	5	51	33	194	110	122	161	198	54	496	398	15	312
**55**	252	330	24	574	644	53	14	3	42	35	210	56	118	121	233	6	448	301	3	296
**56**	183	185	51	369	343	45	11	2	20	30	149	53	41	85	111	22	173	248	11	102
**57**	224	187	116	472	186	71	25	2	53	45	117	71	123	74	323	25	495	230	30	227
**59**	243	235	76	339	200	90	28	10	121	38	153	82	120	145	285	39	507	279	32	204

Sample	*ATM*	*ATR*	*AURKA*	*BACH1*	*BAP1*	*BARD1*	*BRCA1*	*BRCA2*	*BRIP1*	*CHEK2*	*DDB2*	*EMSY*	*FANCD2*	*MRE11*	*NBN*	*PALB2*	*PARP1*	*RAD50*	*RAD51*	*RPA1*
**60**	296	225	134	313	278	134	48	16	96	52	129	91	176	141	278	54	555	278	52	272
**61**	353	275	46	391	288	84	26	12	32	64	133	85	90	168	234	42	516	526	29	308
**62**	269	201	94	332	234	93	36	12	58	44	113	84	153	125	315	46	421	251	42	182
**63**	269	200	207	785	172	152	46	12	90	73	99	95	65	123	220	42	468	393	74	396
**64**	324	273	52	394	365	76	60	11	292	74	391	92	136	179	483	67	354	553	33	261
**65**	325	228	59	592	407	96	46	27	113	46	468	74	258	133	161	58	501	459	44	459
**66**	328	348	90	695	284	54	40	9	49	63	187	65	97	129	311	52	569	553	22	326
**69**	471	340	14	829	537	78	29	2	38	65	401	135	63	235	450	65	486	592	6	368
**70**	265	241	84	312	380	76	54	27	60	61	92	64	132	132	213	46	528	303	38	402
**71**	142	169	49	340	307	58	19	1	50	34	174	39	51	95	141	23	406	211	19	196
**72**	239	245	62	330	319	55	39	11	60	44	201	80	65	124	212	32	422	393	30	182
**73**	297	288	150	407	272	57	62	5	166	45	142	63	134	125	308	37	461	206	40	323
**74**	655	352	6	295	264	10	4	1	25	104	212	51	155	186	147	52	515	939	1	402
**75**	182	206	38	550	213	48	30	7	63	33	160	82	98	67	237	41	541	414	15	268
**77**	347	209	32	409	302	48	29	8	28	34	125	55	81	123	207	26	386	383	7	260
**78**	373	295	31	486	102	78	21	7	98	46	296	115	118	196	320	51	630	393	28	323
**79**	397	220	17	425	131	44	15	9	70	72	268	103	98	189	236	52	262	504	12	228
**80**	287	344	54	486	458	61	46	22	86	52	199	99	81	167	230	25	493	475	27	189
**81**	239	197	26	330	147	37	17	4	51	41	268	79	43	143	199	54	371	474	14	208
**82**	491	255	14	409	322	33	13	12	26	30	279	125	62	247	195	57	364	561	2	191
**83**	302	219	56	667	222	79	47	20	137	68	274	149	100	159	337	57	544	344	30	271
**84**	326	394	3	496	473	40	9	6	43	48	273	90	126	113	336	22	494	305	3	126
**85**	160	184	37	279	366	41	28	6	70	27	87	69	44	74	235	36	514	278	10	119
**86**	441	305	9	524	594	37	10	12	20	62	272	124	84	194	363	61	552	635	8	211
**87**	754	311	18	541	311	58	18	18	68	98	280	176	71	226	432	68	314	573	10	374
**88**	193	279	22	354	505	30	20	2	79	16	257	37	83	157	379	32	416	566	2	183
**89**	233	262	66	359	83	86	59	5	113	78	268	84	147	156	306	46	500	264	42	221
**90**	780	367	32	500	381	59	22	19	149	79	310	129	107	261	389	59	533	473	18	212
**91**	141	413	28	405	167	84	12	5	138	44	464	90	202	154	223	61	312	440	13	280

Sample	*ATM*	*ATR*	*AURKA*	*BACH1*	*BAP1*	*BARD1*	*BRCA1*	*BRCA2*	*BRIP1*	*CHEK2*	*DDB2*	*EMSY*	*FANCD2*	*MRE11*	*NBN*	*PALB2*	*PARP1*	*RAD50*	*RAD51*	*RPA1*
**92**	255	324	21	550	437	58	10	13	50	39	184	68	60	71	202	13	226	413	18	231
**93**	289	299	9	715	481	49	3	3	12	12	322	65	49	128	233	19	263	322	3	293
**94**	404	397	25	557	408	38	17	17	19	60	502	111	63	224	249	60	500	701	4	284
**95**	333	292	15	620	404	49	9	5	42	24	190	86	39	140	176	20	381	491	8	273
**96**	350	305	14	276	103	36	8	3	24	48	424	48	55	153	172	49	299	676	6	262
**MRC-5, P10**	191	155	431	466	233	164	82	36	250	70	125	112	121	153	174	48	639	191	138	541
**MSTO-211H, P6**	72	109	453	135	159	37	110	24	120	45	119	67	153	144	147	41	784	144	46	548
**NCI H2052, P5**	193	138	764	337	248	147	107	43	346	42	676	131	176	365	362	55	700	378	110	564
**NCI H2452, P5**	118	158	338	329	117	126	84	161	109	208	71	77	155	156	204	53	349	328	79	536
