# Supplementary material for: Gene expression profiling of homologous recombination repair pathway indicates susceptibility for olaparib treatment in malignant pleural mesothelioma in vitro
Source: BMC Cancer. 2019 Jan 30;19:108. doi: 10.1186/s12885-019-5314-0 (PMC6354412; doi:10.1186/s12885-019-5314-0)
Supplement: Supplementary file 2 — Figure S1. Expression of tested genes is independent of histomorphology of MPM. The boxplot shows no significant differences between gene expression patterns due to biphasic (B), epithelioid (E), or sarcomatoid (S) MPM. (PDF 59 kb) [file 12885_2019_5314_MOESM2_ESM.pdf]

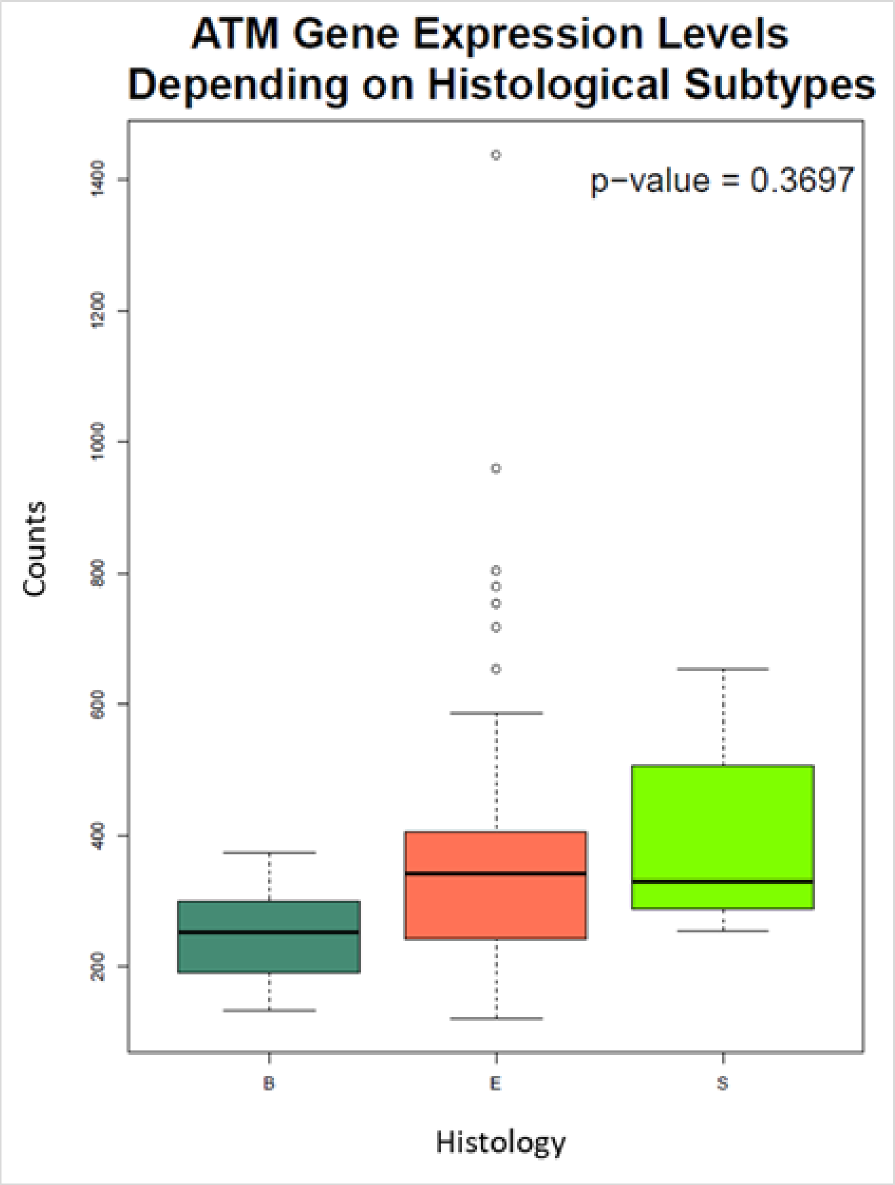

Suppl. Figure1: Expression of tested genes is independent of histomorphology of MPM. The boxplot shows no significant differences between gene expression patterns due to biphasic (B), epithelioid (E), or sarcomatoid (S) MPM.
