# Supplementary material for: Gene expression profiling of homologous recombination repair pathway indicates susceptibility for olaparib treatment in malignant pleural mesothelioma in vitro
Source: BMC Cancer. 2019 Jan 30;19:108. doi: 10.1186/s12885-019-5314-0 (PMC6354412; doi:10.1186/s12885-019-5314-0)
Supplement: Supplementary file 3 — Table S2. AURKA, RAD50, and DDB2 showed statistically significant dependencies on overall and progression-free survival. (PDF 33 kb) [file 12885_2019_5314_MOESM3_ESM.pdf]

Suppl. Table 2: AURKA, RAD50, and DDB2 showed statistically significant dependencies on overall and progression-free survival.

Gene	Likelyhood Ratio Test	Score (logrank) Test	Wald Test	Adjusted p-Values for Likelyhood Ratio Test	Adjusted p-Values for Score Logrank Test	Adjusted p-Values for Wald Test	Hazard Ratio
***AURKA* dependent Overall Survival**	0.00012	1.2E-05	1.6E-05	0.00240	0.00024	0.00032	2.397
***RAD50* dependent Overall Survival**	0.00042	7.5E-05	8.1E-04	0.0042	0.0075	0.0081	2.256
***DDB2* dependent Overall Survival**	0.0019	2.6E-03	2.8E-03	0.0216	0.0135	0.0135	4.373
***AURKA* depended Progression-free Survival**	0.00015	6.5E-05	0.00011	0.0030	0.0013	0.0022	2.262
